# Bile duct stone formation around a nylon suture after gastrectomy: A case report

**DOI:** 10.1186/1756-0500-6-108

**Published:** 2013-03-22

**Authors:** Chiyo Maeda, Naoyuki Yokoyama, Tetsuya Otani, Tomohiro Katada, Natsuru Sudo, Yoshinobu Ikeno, Fumiaki Matsuura, Akira Iwaya, Toshiyuki Yamazaki, Shirou Kuwabara, Norio Katayanagi

**Affiliations:** 1 Department of Digestive Surgery, Niigata City General Hospital, 463-7 Shumoku, Chuo-ku, Niigata City, Niigata 950-1197, Japan

**Keywords:** Choledocholithiasis, Absorbable suture, Gastrectomy

## Abstract

**Background:**

Many cases of choledocholiths formed around sutures and clips used during cholecystectomy have been reported. We describe a case of gallstone formation around a nylon suture after non-biliary surgery. To the best of our knowledge, this is the first report of such a case.

**Case presentation:**

A 75-year-old Japanese man, who had undergone distal gastrectomy for gastric cancer and reconstruction with the Billroth II method 8 years earlier, presented with gastric discomfort. Abdominal ultrasonography was conducted and we diagnosed cholecysto-choledocholithiasis with dilatation of the intrahepatic bile duct. He underwent cholecystectomy and cholangioduodenostomy for choledocholith removal. Gallstones, which had formed around a nylon suture used during the previous gastrectomy, were found in the bile duct. Sutures of the same material had also been placed on the duodenum. Chemical analysis revealed that the stones were composed of calcium bilirubinate. The patient was discharged on postoperative day 19, and choledocholithiasis has not recurred thus far.

**Conclusion:**

The findings from this case suggest that standard, non-resorbable sutures used in gastrectomy may be associated with the formation of bile duct stones; therefore, absorbable suture material may be required to avert gallstone formation even in the case of gastrectomy.

## Background

Some of the risk factors for gallstones are obesity, female gender, genetics, pregnancy, and surgery for gastric cancer [1-3]. It has also been shown that migration of suture material can cause gallstones [4-6].

In 1897, Homans reported the first case of migration of silk sutures into the common bile duct and formation of gallstones [4]. Thus far, many cases of bile duct stone formation around sutures or end clips used in cholecystectomy have been reported [5,6]. However, the mechanism of bile stone formation is unknown.

Gallstone formation is one of the most common complications after gastric cancer surgery. It was reported that the type of gastrectomy and reconstruction had no significant effect on the incidence of gallstone, but the extent of lymph node dissection was a significant contributing factor [3].

Endoscopic retrograde cholangiopancreatography (ERCP) is the gold standard for the treatment of patients with choledocholithiasis [7]. It is relatively difficult to carry out endoscopic treatment with Roux-en-Y anastomosis and the Billroth II method because of the surgically altered anatomy [8]. The success rate of ERCP using the Billroth II method is lower in patients with choledocholithiasis (63–93%) than in patients with an intact stomach (87–97%) [9]. A single- or a double-balloon endoscope is considered useful for such patients [7,10,11]. However, endoscopic procedures are still difficult to perform; they need to be performed by highly experienced endoscopists and require the use of special equipment [9-12]. In cases where ERCP is not successful, a surgical approach may be required.

Herein, we describe a rare case of common bile duct stone formation around a nylon suture after non-biliary surgery, namely, gastrectomy.

## Case presentation

**Figure 1:**
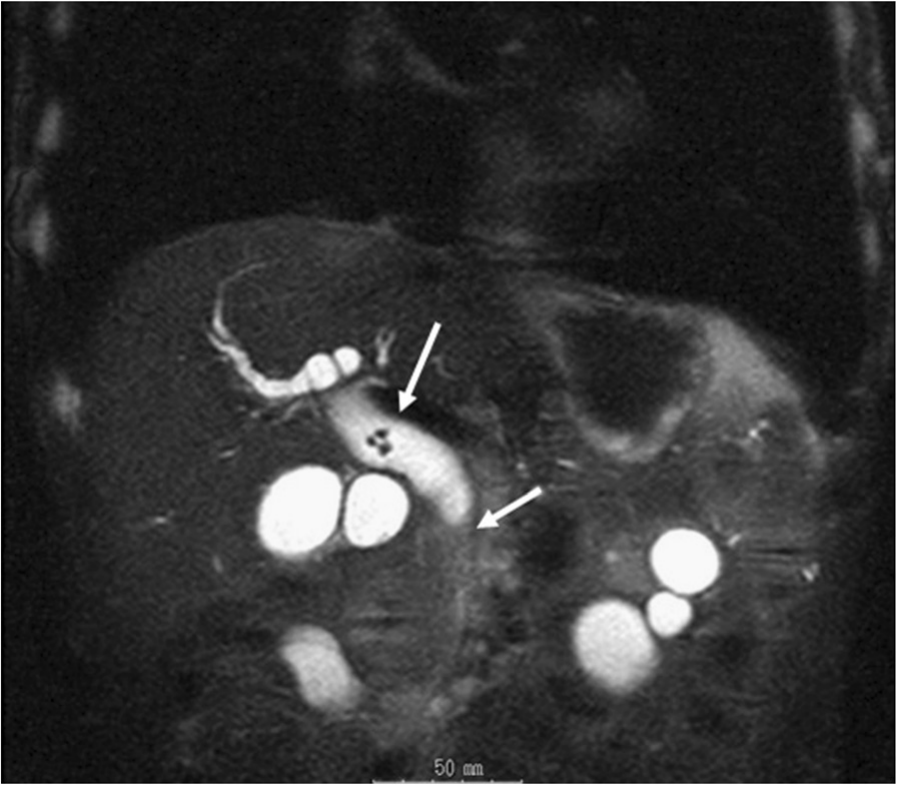
**Abdominal magnetic resonance image (T2) taken at the time of admission.** The arrows indicate bile duct stones.

A 75-year-old Japanese man with a 1-month history of epigastric discomfort was referred to our department. He had undergone gastrectomy with D1 lymphadenectomy for gastric cancer 8 years earlier. Laboratory data revealed hepatic dysfunction. The results of laboratory tests were as follows: total bilirubin, 0.3 mg/dL; glutamate-oxaloacetate transaminase, 341 IU/L; glutamate-pyruvate transaminase, 301 IU/L; gamma-glutamyltranspeptidase, 861 IU/L; and alkaline phosphatase, 1093 IU/L. Abdominal ultrasonography showed cholecysto-choledocholithiasis with dilatation of the intrahepatic bile duct. Computed tomography and magnetic resonance imaging also showed gallbladder and bile duct stones (Figure 1). ERCP was not performed because the patient’s stomach had been reconstructed using the Billroth II method and because of non-availability of an experienced endoscopist and the required equipment in our hospital. Based on our findings, we concluded that cholecystectomy and cholangioduodenostomy were appropriate for this patient.

**Figure 2:**
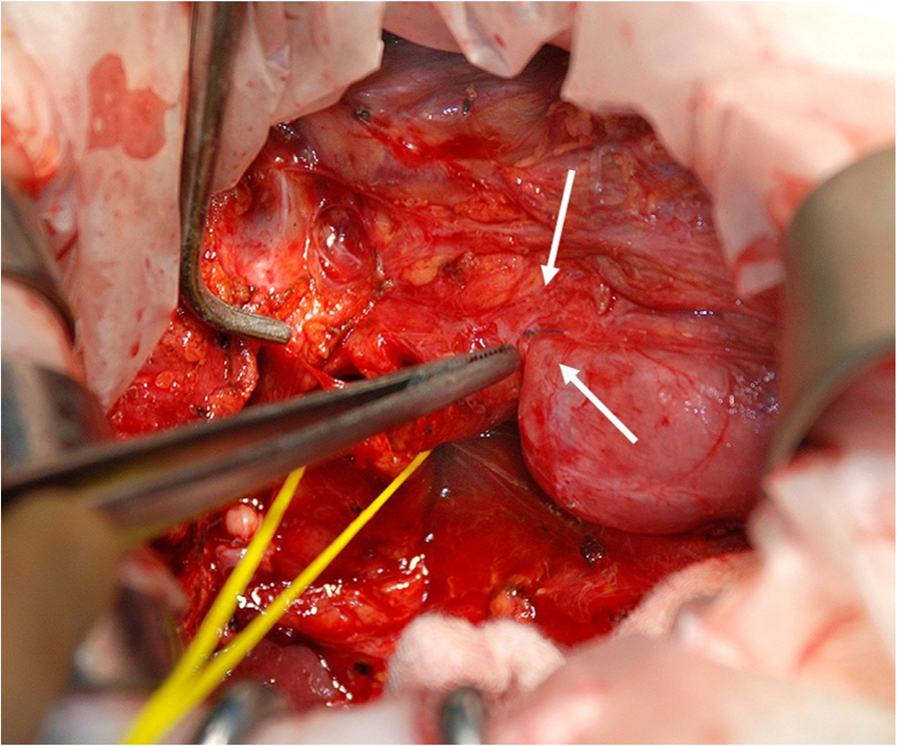
The nylon sutures used during an earlier gastrectomy placed around the duodenum.

**Figure 3:**
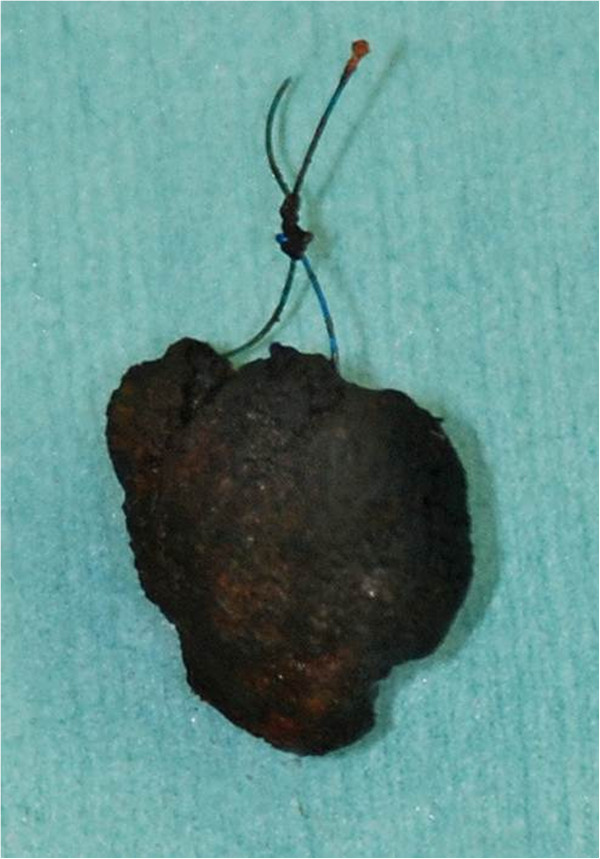
A calcium bilirubinate stone containing a nylon suture.

Under general anesthesia, a laparotomy was performed via a right subcostal incision. Although adhesion around the bile duct was not strong, the dilated common bile duct was compressed by surrounding tissues. Several nylon sutures used during the earlier gastrectomy of the duodenum were identified (Figure 2). A small incision was made into the common bile duct. Five stones in the bile duct were extracted, using an intraoperative cholangioscope. The stone at the bottom of the common bile duct contained the tip of a nylon suture, which was made of the same material as the sutures on the duodenum (Figure 3). After the stones were extracted, cholecystectomy and cholangioduodenostomy were performed.

The patient was discharged on postoperative day 19. His postoperative course was uneventful, and he recovered without any complications. Choledocholithiasis has not recurred after the surgery.

Chemical analysis revealed that the stones were composed of calcium bilirubinate and fatty acid calcium. Microscopic examination revealed chronic cholecystitis with formation of Rokitansky-Aschoff sinuses.

## Conclusions

The formation of gallstones is thought to be multifactorial, but obesity, genetics, pregnancy, and gastric surgery have been found to be predisposing factors; women are also thought to be more predisposed to gallstones than men [1-3]. In addition, suture materials that have migrated to another part of the body are known to act as a nidus for stone formation after biliary surgery, although the mechanism underlying this is unknown [12-14]. We have presented here a rare case of bile duct stone formation around a suture used during gastrectomy. In this patient, not only gastrectomy but also migration of a suture was considered as the causative factor for stone formation.

One hypothesis stipulates that chronic compression by surrounding tissues may result in the formation of bile duct stones after laparoscopic cholecystectomy. Kitamura et al. suggested that when the clipped cystic duct is compressed by surrounding tissues, the duct is inverted into the lumen of the common bile duct, and finally, the necrotic stump of the cystic duct is covered by the surrounding tissues [12,15]. This migration allows bile crystals to adhere to the foreign body. Another hypothesis suggests that choledocholithiasis may be caused by bile leakage [6,15]. The clip may be improperly applied during surgery, leading to incomplete closure of the cystic duct and allowing for the development of a biloma. When this biloma later drains off into the common bile duct via the cystic duct, the clip also migrates into the common bile duct. However, as Tsumura et al. showed, in 75% cases, the clips migrate without bile leakage [16]. This latter hypothesis does not apply to our patient. Our patient had undergone gastrectomy, a non-biliary surgery; therefore, our findings may be explained using the first hypothesis. We believe that the surrounding tissues compressed the common bile duct, resulting in ischemic parietal damage at the site of chronic compression. This process likely allowed the nylon suture to migrate into the bile duct.

It has been suggested that absorbable suture material be used for biliary tract surgery [6,12,13,17]. However, absorbable material can also act as a nucleus for subsequent bile duct stone formation until it is absorbed [5,18]. In the case of laparoscopic cholecystectomy, Jain et al. have recommended the use of an ultrasonically activated scalpel instead of clips to decrease the chances of morbidity [19]. Tsumura et al. revealed that the period between laparoscopic cholecystectomy and appearance of the first symptoms of clip migration ranged from 7 to 17 months [6,16]. In our patient, although the time of stone formation is unclear, gallstone formation may have been avoided if absorbable sutures had been used during the surgery.

To the best of our knowledge, this is the first report of a case of choledocholithiasis in which the stone formed around a suture used during gastrectomy. We therefore suggest that absorbable suture materials be used even in gastrectomy to prevent gallstone formation.

## Consent

Written informed consent was obtained from the patient for publication of this case report and any accompanying images. A copy of the written consent is available for review by the Series Editor of this journal.

## Competing interests

The authors declare that they have no competing interests. No financial support was received.

## Authors’ contributions

CM was the major contributor to writing the manuscript. NK and TO revised the manuscript and provided the final approval of the version to be published. TK, NS, YI and FM were involved in the patient’s medical care. AI and TY were responsible for drafting the manuscript. SK and NK reviewed the manuscript. All the authors have read and approved the final manuscript.
